# Correction to “Immune Tolerance Induction With a Recombinant Factor VIII Fc in Haemophilia A: Data From a Chart Review Study”

**DOI:** 10.1111/ejh.70043

**Published:** 2025-10-15

R. Klamroth, M. Al Saleh, H. Glosli, M. Schiavulli, B. Guillet, L. Bystrická, A. Schönstein, and S. Lethagen, “Immune Tolerance Induction With a Recombinant Factor VIII Fc in Haemophilia A: Data From a Chart Review Study,” *European Journal of Haematology* 115 (2025): 134–141, https://doi.org/10.1111/ejh.14427.

The second sentence of Paragraph 3.4.2 | Rescue ITI of the Safety section (3.4): “Seven (41.2%) of these patients had SAEs, and one (5.9%) event was adjudicated as a serious adverse drug reaction by the investigator (Table S5).” was incorrect. This should have read: “Seven (41.2%) of these patients had SAE. One (5.9%) SAE was assigned as a serious adverse drug reaction (SADR) by the Sponsor based on predefined conservative criteria, as relatedness was not assigned by the Investigator (Table S5).”

The last row of Table S5 (“SAEs and SADRs in rescue ITI patients”) in the Supplementary Appendix and its footnote was incorrect, as the last listed SAE (Under “Vascular disorders”, i.e. Haematoma) was also classified as a SADR by the Sponsor based on predefined conservative criteria, with relatedness not assigned by the Investigator:

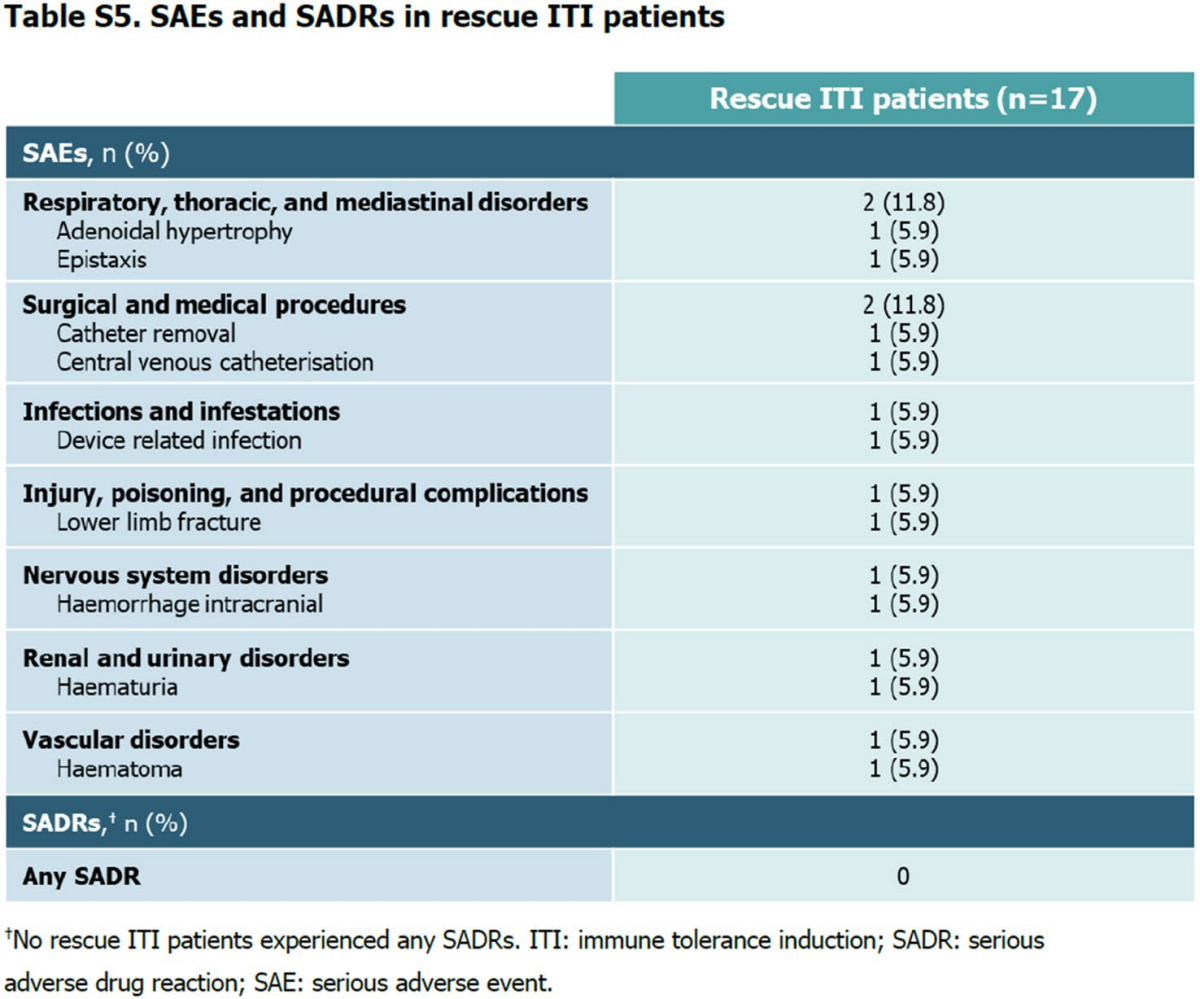

The last row of the table and respective footnote was corrected accordingly:

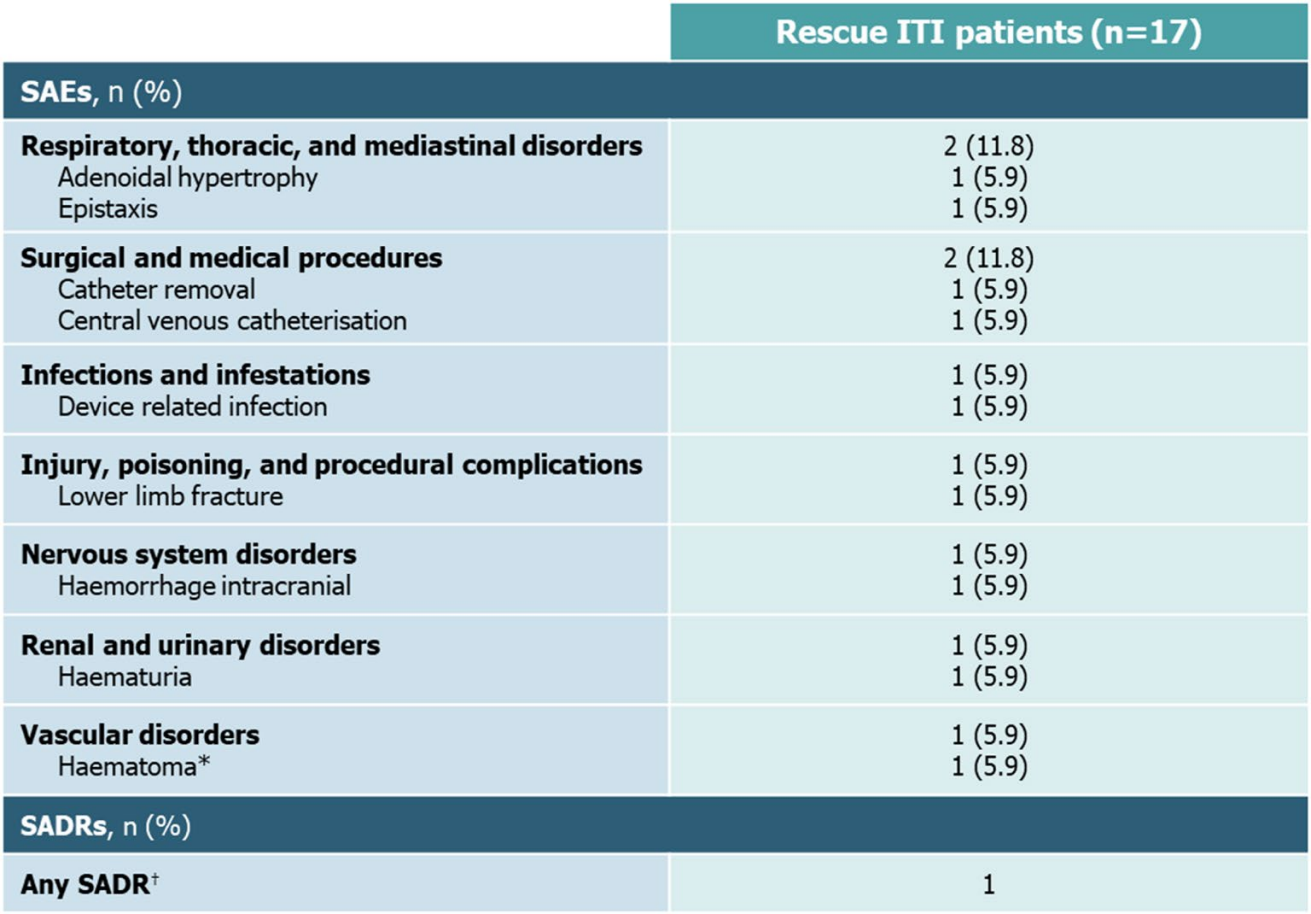

*This SAE (haematoma) was also classified as a SADR (^†^) by the Sponsor based on predefined conservative criteria, as relatedness was not assigned by the Investigator. ITI: immune tolerance induction; SADR: serious adverse drug reaction; SAE: serious adverse event.

The conclusions of the paper are not affected. We apologize for these errors.

